# A roadmap to achieve pharmacological precision medicine in diabetes

**DOI:** 10.1007/s00125-022-05732-3

**Published:** 2022-06-24

**Authors:** Jose C. Florez, Ewan R. Pearson

**Affiliations:** 1 Center for Genomic Medicine and Diabetes Unit, Department of Medicine, Massachusetts General Hospital, Boston, MA USA; 2 Department of Medicine, Harvard Medical School, Boston, MA USA; 3 Programs in Metabolism and Medical & Population Genetics, Broad Institute of Harvard & MIT, Cambridge, MA USA; 4 Department of Population Health & Genomics, School of Medicine, University of Dundee, Dundee, Scotland UK

**Keywords:** Biomarker, Diabetes, Genetic, Monogenic, Personalised medicine, Pharmacogenetics, Pharmacological, Precision medicine, Review, Treatment

## Abstract

Current pharmacological treatment of diabetes is largely algorithmic. Other than for cardiovascular disease or renal disease, where sodium–glucose cotransporter 2 inhibitors and/or glucagon-like peptide-1 receptor agonists are indicated, the choice of treatment is based upon overall risks of harm or side effect and cost, and not on probable benefit. Here we argue that a more precise approach to treatment choice is necessary to maximise benefit and minimise harm from existing diabetes therapies. We propose a roadmap to achieve precision medicine as standard of care, to discuss current progress in relation to monogenic diabetes and type 2 diabetes, and to determine what additional work is required. The first step is to identify robust and reliable genetic predictors of response, recognising that genotype is static over time and provides the skeleton upon which modifiers such as clinical phenotype and metabolic biomarkers can be overlaid. The second step is to identify these metabolic biomarkers (e.g. beta cell function, insulin sensitivity, BMI, liver fat, metabolite profile), which capture the metabolic state at the point of prescribing and may have a large impact on drug response. Third, we need to show that predictions that utilise these genetic and metabolic biomarkers improve therapeutic outcomes for patients, and fourth, that this is cost-effective. Finally, these biomarkers and prediction models need to be embedded in clinical care systems to enable effective and equitable clinical implementation. Whilst this roadmap is largely complete for monogenic diabetes, we still have considerable work to do to implement this for type 2 diabetes. Increasing collaborations, including with industry, and access to clinical trial data should enable progress to implementation of precision treatment in type 2 diabetes in the near future.

**Graphical abstract:**

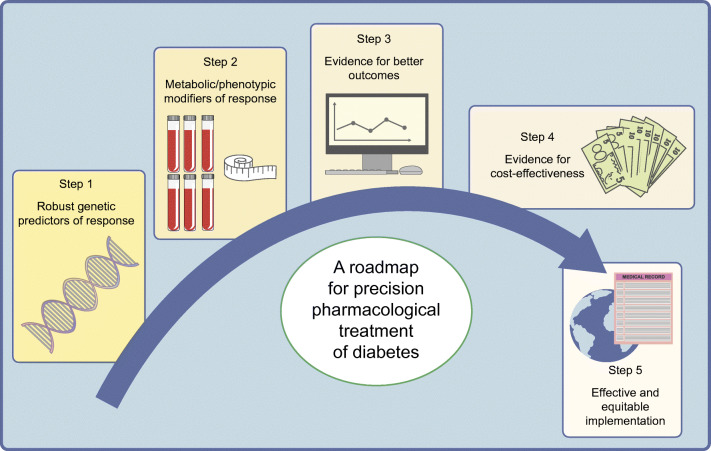

**Supplementary Information:**

The online version contains a slideset of the figures for download, which is available at 10.1007/s00125-022-05732-3.

## Current context

The treatment of type 2 diabetes is currently algorithmic. Professional guidelines elaborated by expert panels typically recommend initiation of pharmacotherapy with metformin, a biguanide that lowers blood glucose in a variety of insulin-independent mechanisms [1] and has been shown to be safe, cheap and effective. For those at high risk for, or with pre-existing, cardiovascular disease, or with proteinuric kidney disease, there is now strong evidence for treatment escalation with sodium–glucose cotransporter 2 (SGLT2) inhibitors and/or glucagon-like peptide-1 receptor agonists (GLP-1RAs). However, for the majority, escalation of treatment is triggered by failure to meet glycaemic targets, and involves the incorporation of second- and third-line agents to the treatment regimen. Of the 12 drug classes currently available to practitioners, the decision on which agent to add is usually predicated on considerations such as the patient’s comorbidities, the desire to avoid specific side effects, cost or availability. Critically, these choices are never made by a principled evaluation of the specific pathophysiological processes that caused the patient’s hyperglycaemia in the first place: though a weight-losing medication may be selected in someone with obesity and concomitant insulin resistance, the decision is reached on a clinical assumption rather than on a rigorous ascertainment of the molecular or physiological defects that led to the person’s current metabolic state.

This empiric approach stands in stark contrast with emerging trends in other areas of medicine. In oncology, older chemotherapeutic regimens that would inhibit cellular proliferation non-specifically and were designed on the basis of clinical trials centred on a particular anatomical origin of the primary tumour have been largely replaced by biological and chemical agents targeting the specific disruptive mutations that alter the cellular growth programme identified in the tumour sample. Pharmacological approaches to autoimmune diseases have experienced a qualitative leap driven by the elucidation of the pathways affected in each clinical condition, such that biological agents are now prescribed to modulate the immunological defect that causes the clinical syndrome. In cardiology, prevention of coronary artery disease (CAD) addresses each of the mechanisms (hypercholesterolemia, platelet aggregation, hypertension) that contributes to atherosclerotic thrombotic events.

Therefore, the question that arises is whether the current approach for type 2 diabetes management is adequate. We believe it is not, based on three key considerations:

1. The epidemiological evidence suggests that we are far away from reaching glycaemic targets [2]. Though there are multiple potential reasons for this failure (e.g. cost, access, adherence, education, etc.), it is quite possible that the medication(s) being prescribed may not be optimal for each individual patient.
2. In support of the last assertion, evidence from multiple clinical trials indicates that there is great heterogeneity in the human response to particular therapeutic modalities, with a substantial number of participants failing monotherapy over time [3, 4].
3. Most significantly, none of the currently available options ‘cures’ diabetes or causes remission: unless a significant lifestyle change ensues, preferably early in the disease process and resulting in substantial weight loss (of the type only achieved after bariatric surgery or after very low calorie diet [5]), the patient with type 2 diabetes remains on pharmacotherapy for life, his/her pathophysiology continues unabated and the typical course involves escalation of therapy.

We therefore postulate that a promising avenue to achieve better control of the diabetes pandemic, and potentially reverse the disease process, is to advance a change in the treatment paradigm that deploys our therapeutic armamentarium in the metabolic context most likely to succeed. This requires (1) determining the predominant mechanism that led to each individual’s diabetes; (2) elucidating his/her current metabolic state; and (3) understanding the mode of action of the available drugs, such that the therapeutic choice is mainly driven by the underlying biology, in hopes of correcting it.

## A vision for the future: the ideal scenario

What does that scenario look like, in real life? By re-imagining the ideal clinical encounter, we can highlight what needs to happen for this vision to be realised, and how realistic it is to achieve it in the short- to medium-term given the present state of the field.

To determine the predominant mechanism that led to each individual’s diabetes, we first need to achieve much greater granularity in the diagnostic process. As is outlined in other contributions to this issue on precision diabetes, the current classification of diabetes types is imperfect. In particular, type 2 diabetes is mainly a diagnosis of exclusion: any hyperglycaemia that is not caused by a single-gene mutation, an established genetic syndrome, autoimmunity or a secondary pancreatic defect is labelled as ‘type 2 diabetes’. This is because the diagnosis is established simply by crossing a glycaemic threshold: since hyperglycaemia is the common end result of many disparate pathophysiological processes, the diagnosis of type 2 diabetes is really a ‘grab basket’ of many different conditions, and this heterogeneity hampers the selection of tailored pharmacotherapeutic regimens.

Recent evidence described elsewhere in this issue suggests that type 2 diabetes can be parsed into distinguishable, even if partially overlapping, subtypes [6–9]. One approach involves the use of genetically anchored but physiologically informed clusters, in the form of process-specific or partitioned polygenic scores (PSs), composed of a subset of genetic variants associated with specific biological processes [7, 10]. This strategy has the advantage that genetic information only needs to be obtained once in the lifetime of the individual and is not affected by the disease itself or by its treatment. One drawback is that taken in isolation it neglects the contribution of environmental or behavioural factors that modify or interact with the genetic risk.

An alternative approach involves the use of phenotypic characteristics alone, in that some of these metrics (e.g. fasting glucose, BMI) capture both genetic and environmental information, and do not require the generation of genomic data in the entire population which may well be a barrier to implementation in many healthcare systems. Many of these phenotypic variables are routinely captured in clinical management of type 2 diabetes, including in low-resource healthcare systems, and as such offer a readily tractable route to precision medicine without the need for genetic or other molecular characterisation of patients. An evident limitation is that both the disease process and its treatment can influence many of these variables, and thus paying attention to the metabolic and developmental state of the person along his/her disease course may be critical in their interpretation.

Nevertheless, we propose that in our ideal scenario genetic data can serve as an informative starting point. In a future world where every individual has their genome sequenced or is genotyped as soon as they enter the healthcare system (a not unrealistic prospect, given current favourable trends in the cost of genotyping or sequencing technologies), the moment somebody is diagnosed with diabetes by a hyperglycaemic blood reading, pre-existing genetic information deposited into the medical record would produce a preliminary estimate of the genetic burden that has predisposed that individual to develop diabetes. It is possible that such an assessment may indicate a most likely or predominant mechanism in a fraction of the cases, currently estimated in 25–30% of people with type 2 diabetes [7]; however, the majority of cases may have type 2 diabetes caused by a constellation of factors that could make it difficult to place individuals into discrete categories [11]. In this ‘palette’ model, where each person represents a specific hue composed of multiple primary colours, using quantitative scales for each of the factors that assign each person an aggregate risk score may hold greater power and be extensible to more individuals [8], although in the end arbitrary thresholds may need to be adopted to facilitate dichotomous clinical decision making. In either case, that initial genetically based assessment would then be refined by a clinician-triggered measurement of biomarkers at the time of diagnosis, which would confirm, refine or modify the diabetes subtype or aggregate risk score suggested by the genetic algorithm. Robust clinical trial evidence would have already demonstrated that assigning a diabetes subtype or aggregate risk score to the patient might alter the therapeutic and/or surveillance strategy, and would lead to improved patient outcomes. That information would be available to the clinician at the point of care, such that when a diabetes subtype is confirmed for the patient a tailored pharmacological regimen and a programme for interval monitoring for complications can be adopted in real time.

For this ideal vision to be realised, we need (1) genome-wide genetic information included into the medical record; (2) solid evidence supporting that certain genetic profiles predict differential response to specific drug classes; (3) dynamic metabolic modifiers of those static genetic predictors to become easily available in the clinic; (4) outcomes evidence suggesting that such an approach leads to better clinical outcomes and is more cost-effective; and (5) a translational strategy that allows for this complex information to be implemented at the point of care, so that the clinician can seamlessly access the decision tool as soon as hyperglycaemia has been documented.

Such a picture is not science fiction: it has already been adopted in the context of monogenic diabetes. When a person presents clinically with neonatal diabetes (diabetes that develops in the first 6 months of life) or MODY (autosomal dominant familial diabetes in a young and lean person, with a non-ketotic and antibody-negative presentation), the diagnosis triggers a genetic test for the specific molecular subtype, and the result determines whether the patient can be transitioned from insulin to high-dose sulfonylurea (in the case of neonatal diabetes) [12, 13], from metformin or insulin to a low-dose sulfonylurea (in the case of *HNF1A*-MODY) [14, 15] or to no pharmacological therapy at all (in the case of *GCK*-MODY) [16]. It is not utopian to assume that what has already become the standard of care in monogenic diabetes could, in the not-too-distant future, be adopted to some extent in polygenic diabetes, in cases where the genetic and metabolic tools carry a comparable risk prediction profile and are associated with similar levels of evidence supporting favourable clinical outcomes.

**Fig. 1:**
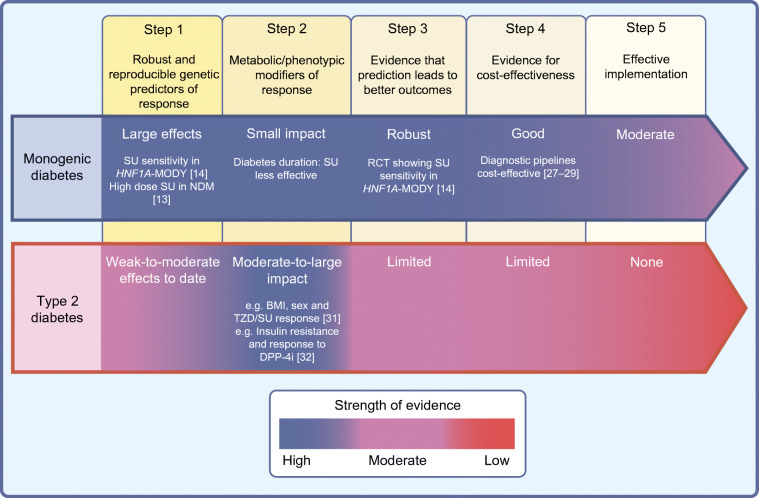
A roadmap to achieve pharmacological precision medicine in diabetes. Steps 1 to 5 describe the necessary steps for discovery, validation and implementation of precision medicine approaches to the management of diabetes. This is depicted for monogenic diabetes and type 2 diabetes. The colour represents the current strength of evidence, with blue being high and red being low. DPP-4i, DPP-4 inhibitor; NDM, neonatal diabetes mellitus; SU, sulfonylurea; TZD, thiazolidinedione. This figure is available as part of a downloadable slideset

In the sections that follow, we state what we believe is needed for each of these components to emerge, and describe how far along we are towards reaching that goal. These steps are outlined in Fig. 1.

## What is needed?

### 1. Robust and reproducible genetic predictors of response

The field of pharmacogenetics in diabetes is still in its infancy, but a general picture of the genetic architecture of the glycaemic response to antidiabetic drugs is beginning to coalesce. Genome-wide association studies (GWAS) for metformin [17, 18] and sulfonylureas [19] numbering in the several thousand samples have identified a handful of loci associated with drug response (in or near the genes *ATM* and *SLC2A2* for metformin, and *GXYLT1* and *SLCO1B1* for sulfonylureas). More importantly, these GWAS datasets have not revealed genetic associations of large effects, whilst assessments of heritability [19, 20] have shown that these traits are similar to other complex traits, in that multiple genetic variants and environmental factors contribute in concert to the observed phenotype.

Given these findings, the quest for individual genetic associations will require much larger samples, including from non-European ethnicity, only achievable via international collaboration. Whilst this global effort may yield insights that help illuminate the molecular mode of action of specific drugs, it is unlikely to produce individual genetic variants that harbour strong predictive power at the population level. Therefore, the attention has recently shifted to the construction of PSs, composed of multiple variants, which in aggregate explain a greater proportion of the variance observed.

The construction of such scores can follow a number of avenues. Investigators could focus on genes that encode the enzymes responsible for metabolising the relevant drug, under the assumption that pharmacokinetic parameters have the greatest relevance on circulating drug levels and subsequent clinical effects. To identify the variants that would populate such PSs, studies that incorporate measurements of the drug and its metabolites at specific time points are needed, but unfortunately these tend to be limited in size. An alternative is to develop PSs in which genetic variants are clustered according to their impact on specific metabolic traits, in an attempt to group them along physiological pathways: these process-specific or partitioned PSs, referenced above and elsewhere in this issue, have been recently developed for type 2 diabetes [7, 10]; whether individuals at one extreme of the genetic risk for each of these subtypes of type 2 diabetes experience a differential response to specific pharmacological therapies is a subject of active investigation. PSs for drug response can also be constructed from GWAS designed specifically with that endpoint in mind, and they can include only the variants that reach genome-wide significance (defined at *p*<5 × 10^−8^), termed restricted to significant PSs (rsPSs); or they can include a much larger set of variants below that stringent statistical threshold, under the assumption that a large number of false negative associations lie beneath the cutoff and will improve predictive power, in globally expanded PSs (gePSs). In either situation, many thousands of samples are needed to generate robust and reproducible associations, and these are presently only available for a handful of type 2 diabetes drugs [17–19]. Finally, it should be noted that whilst glycaemic endpoints have been traditionally used as outcomes in these investigations, there is increased interest in other clinical endpoints of greater relevance to patients, such as those having to do with the cardiovascular system (e.g. CAD, congestive heart failure or stroke), diabetic kidney disease or retinopathy.

At the time of writing, beyond the aforementioned GWAS for metformin and sulfonylureas examining glycaemic outcomes, there is a similar ongoing effort for GLP-1RAs led by the Metformin Genetics Plus Consortium, which is composed of international partners with access to retrospective pharmacogenetic datasets, most frequently centred on the electronic health record (EHR). A new Consortium seeking to gather clinical trial data has also been formed as a joint venture between Canadian and UK partners. This initiative between the University of Dundee (E. R. Pearson) and Montreal Heart Institute (M.-P. Dubé) has obtained GWAS on clinical trial data for glycaemic response and cardiovascular outcomes for the novel type 2 diabetes drug classes GLP-1RAs, SGLT2 inhibitors and dipeptidyl peptidase-4 (DPP-4) inhibitors, and should report in the next 12 months (see https://gtr.ukri.org/projects?ref=MR%2FT032014%2F1). The National Institutes of Health (NIH)-funded Glycemia Reduction Approaches in Diabetes: A Comparative Effectiveness Study (GRADE, see https://grade.bsc.gwu.edu) is a comparative effectiveness trial in which just over 5000 participants with type 2 diabetes on metformin therapy were randomised to one of four arms (glimepiride, sitagliptin, liraglutide and insulin glargine); a GWAS is currently being conducted in this dataset. It is hoped that as the results from these various studies become available the PS to be produced will acquire greater predictive power.

### 2. Robust and reproducible metabolic or phenotypic biomarkers

It is critical to note that whilst germline genetic information harbours the distinct advantage of immutability in the lifetime of an individual and thereby preservation from reverse confounding, it is necessarily static. In the complex trait world, where genetic variation only explains one component of the phenotype and often interacts with the environment, genetic prediction can only be probabilistic, in contrast to the deterministic assessments often encountered for Mendelian traits. As such, a person with the same genetic profile from birth may respond differently to exogenous stimuli depending on a variety of factors, including his/her current metabolic state [11, 21]. This has been noted, for instance, in the differential response to sulfonylureas seen over time in individuals classified as having ‘severe insulin deficient diabetes’ [6], whereby an initial favourable response waned relatively quickly as the individual beta cell reserve presumably was more rapidly exhausted in this group [8].

Therefore, any genetic predictors that derive from the well-powered genomic studies described above will need to be complemented with biomarkers that capture orthogonal information and in essence ‘actualise’ genetic predictions. These might include measures of adiposity, disease duration, beta cell function or insulin action: in this regard, a basal or stimulated C-peptide holds singular promise. In addition, agnostic explorations for such biomarkers as can be assessed by global metabolomic profiling are of intense interest [22–24].

### 3. Evidence that such prediction leads to better clinical outcomes

Even if the genetic determinants of drug response and the molecular biomarkers that refine this prediction are identified, we must ensure that obtaining such information and acting on it improves clinical outcomes. It could very well be that whilst the ability for such an instrument to predict the outcome is statistically significant, the magnitude of the effect is not clinically relevant, or adds only a limited amount to simple clinical measures. For example, if a PS associated with metformin response is used to stratify the population into extremes of ‘top responders’ and ‘response failures’, but the contrast between the two groups only amounts to a difference in HbA_1c_ of 0.1%, it is unlikely that clinical decision making would be affected by such an instrument, as the downstream impact on diabetic complications would be minimal.

To assess the magnitude of these effects, investigators need access to clinical trial data. Unfortunately, the majority of clinical trials for novel type 2 diabetes drugs have been conducted by the pharmaceutical industry. Barriers to accessing these results include the proprietary nature of industry-sponsored clinical trials, the boundaries imposed by informed consent obtained at the time of trial design, the potential identifiability afforded by genetic information, data protection measures enacted by governmental and regulatory authorities, and the logistic hurdles involved in assembling and disseminating such information. Nevertheless, there are several precedents where forward-looking pharmaceutical companies have collaborated with academic and/or government scientists in advancing valuable knowledge in the pre-competitive space. In the type 2 diabetes field, the Innovative Medicines Initiative (IMI) funded by a European Union public–private partnership has supported a variety of type 2 diabetes-related projects: the IMI-DIRECT Consortium (https://www.imi.europa.eu/projects-results/project-factsheets/direct) was specifically designed to advance precision medicine in diabetes, and included a pharmacogenetic component [25]. Its goals have been largely assumed by the subsequently formed IMI-RHAPSODY Consortium (https://www.imi.europa.eu/projects-results/project-factsheets/rhapsody). Similarly, the Accelerating Medicines Partnership (AMP) in the USA convenes the NIH, academic investigators and pharma partners to elucidate molecular mechanisms and advance the identification of drug targets, and has focused on type 2 diabetes (AMP-T2D) and common metabolic diseases (AMP-CMD) as areas of specific interest. Under AMP-T2D (https://www.nih.gov/research-training/accelerating-medicines-partnership-amp/type-2-diabetes), it supported the generation of the largest whole-exome sequencing dataset for type 2 diabetes [26]; under AMP-CMD (https://www.nih.gov/research-training/accelerating-medicines-partnership-amp/common-metabolic-diseases), it supports the AMP-CMD Knowledge Portal (https://hugeamp.org), which contains genomic and metagenomic datasets on ~350 metabolic traits available for mining and exploration, as well as a number of functional genomics projects to advance from genetic association to molecular function. A valuable byproduct of these initiatives is the increased collaboration and interaction between academic and industry investigators, and it is hoped that access to pharmaceutical clinical trial data may ensue as a reasonable aspiration.

An alternative to clinical trial data attained via industry–academic collaborations is to delve into the EHR, where a plethora of clinical data (often of a longitudinal nature) are available and are increasingly amenable to investigation via machine learning and artificial intelligence approaches. Several health system-based biobanks have been created in academic medical centres, which have invested resources to obtain general consent from participants as well as genome-wide DNA data. In some cases, entire national jurisdictions have done likewise, and programmes like the UK Biobank, FinnGen, the Estonian Biobank, the US Million Veterans Program, the US All Of Us Research Program, Biobank Japan, the China Kadoori Biobank and many others are already yielding results. These widely available datasets can be accessed to test whether predictive tools are associated with clinical outcomes, and whilst the initial focus might be on glycaemic measures it is possible to assess a multitude of other relevant endpoints.

It is also important to point out that there is a need to greatly increase the genetic and phenotypic studies of drug response in non-Europeans, whether in trial datasets or biobanks. Variants and indeed PSs developed in European populations may have limited effect in non-Europeans, who may have ethnicity-specific variants that are relevant to their treatment.

A note about causality is warranted here. A common criticism of studies that leverage pre-existing clinical trials, cohorts or biobanks is that any observed association merely denotes a correlation, and is not free of the confounding and biases inherent to retrospective studies. The corollary of this assertion is that no causality can be inferred until an appropriately designed prospective, randomised clinical trial is conducted to assess whether a genetic instrument truly predicts an outcome—an insurmountable and unaffordable task for the length of time required to carry out such trials across multiple phenotypes for multiple biomarkers. However, it should be noted that genetic predictors are unique among biomarkers in three key respects: indeed, (1) germline DNA sequence variation is present from the moment of conception, i.e. before the onset of any phenotype, and thus the arrow of time is unidirectional; (2) it is inherited in a largely random manner, as maternal and paternal alleles distribute stochastically at meiosis, providing a natural experiment of randomised genetic exposure; and (3) it is immutable through the lifetime of the individual, and thus free of the effects of reverse confounding. Therefore, robust genetic associations contain within them the seed of causal inference, and as such we favour genetic instruments as anchors of nascent clinical predictors.

### 4. Evidence for cost-effectiveness

Investigators may be able to identify genetic determinants of clinical outcomes and refine these predictive instruments with current biomarkers; they may even demonstrate that the predictions made are clinically significant and lead to improved health outcomes. However, if the incorporation of such instruments to the clinical armamentarium is inordinately expensive, it will not be possible to deploy it at scale, and it may contribute to magnifying health disparities. It is therefore imperative that the tools that are developed take both throughput and cost into account.

On the benefit side of the scale, the morbidity and mortality associated with diabetes and its complications is substantial, and the effort spent in surveillance and prevention taxes the most robust economies. Being able to discriminate who is at greatest risk of a given vascular complication, or more likely to respond to a specific therapy, should facilitate an intelligent stewardship of resources. It is easy to envision that great gains may be reaped in this regard.

On the other side, the cost of genomic technologies (genotyping and sequencing) continues to plummet, and it is not too extravagant to assume that it may be possible to obtain genomic information from every individual the moment they enter a healthcare system, or even at birth (although we recognise this is an area of ongoing ethical debate). Such information could be used for multiple diseases and indications, and would pre-empt the need for a disease-specific test every time a new condition arises. Though some advocate for disease-specific genotyping using inexpensive customised arrays, it would seem most efficient to capture the entire genome once with a single lifetime test.

Other relevant biomarkers would be measured at the relevant developmental stage or metabolic state. Here again, sophisticated and complex metabolomic platforms might be used for discovery; however, once a candidate biomarker is selected to complement the genetic information, its measurement must be simple, affordable and scalable if one hopes for precision diabetes to penetrate low- and middle-income countries, where the bulk of people with type 2 diabetes reside. As these tools are developed, the proper cost-effectiveness analyses, using simulations and prospective studies, ought to be carried out. These types of studies have already proven that identifying monogenic diabetes is cost-effective [27–29], and that using metformin to prevent diabetes in people at high risk is cost-saving [30].

### 5. Effective and equitable implementation

Once the theoretical groundwork has been established, the predictive tools have been designed and refined, and their clinical utility and cost-effectiveness have been demonstrated, the vision described above has to be implemented at the point of care. The proposed strategy entails universal genomic ascertainment, through a generic GWAS array, sequencing or disease-informed panels, if not immediately, at least eventually, with disease-specific chips potentially serving as the bridge. This information would be available in the patient’s EHR, ready to be harnessed as various health needs arise.

Algorithms would have been developed to mine the broad swath of genomic data and produce a quantitative assessment of the person’s risk, subtype of diabetes or likelihood to respond to a specific therapy. The clinical trigger for the algorithm to be run might be a blood test meeting the diagnostic threshold for diabetes, or a prescription for a diabetes drug: at that point, the algorithm would reach into the genomic sequence and provide a probabilistic prediction and concomitant interpretation for the clinician. The result may be accompanied by the recommendation to order a specific test, to measure the biomarker(s) that have been shown to modify or refine the genetic prediction. The algorithms and ancillary biochemical evaluations might be updated iteratively as new information emerges and reaches the level of actionable evidence.

In addition to the clinical workforce, appropriate education on the value and use of these predictive tools would need to be disseminated to patients, regulators and payors. As society embraces their adoption, it must do so with a firm commitment to democratisation and generalisability so as to reduce health disparities (see Text box). 
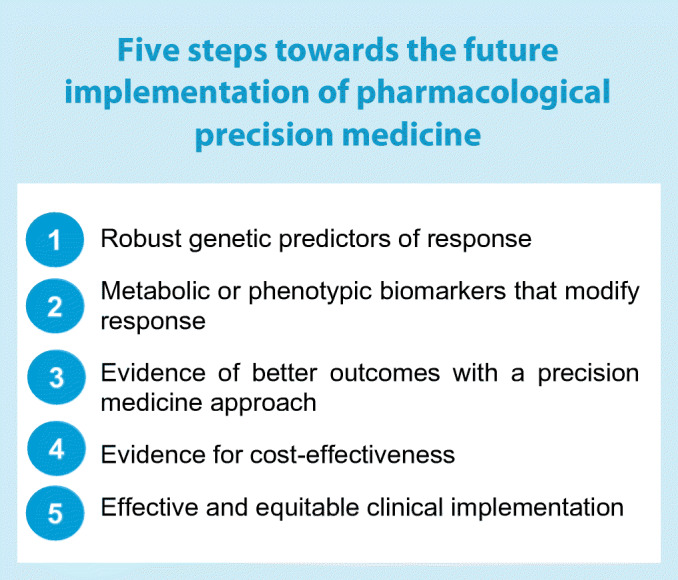


## Caveats: reality vs the ideal

In this perspective, we have proposed a vision for what we consider an ideal approach to achieve pharmacological precision medicine. We outline the steps required to establish that, for type 2 diabetes, such an approach has clinical utility, is cost-effective and can be readily implemented, an approach largely realised for monogenic diabetes. The relative contributions of genotype, molecular biomarkers or clinical measures have yet to be established, and at the end of this process we may conclude that for prediction of drug response, genetics may not add to clinical phenotype or to simple physiological metrics such as measuring C-peptide. But this does not detract from a focus on genetic drivers of diabetes and drug response that provide the invariant causal biological mechanism for variation in drug outcomes. We recognise that our approach may be considered idealistic by some, and acknowledge that in the context of global diabetes, where healthcare costs are a key consideration and where available diabetes drug treatments are limited, a vision focused on genetics and other molecular biomarkers is currently unrealistic. As discussed above, the most tractable approach to implementation of precision medicine in these contexts will be to focus on available clinical measures such as sex and BMI [31, 32]. However, given the falling costs of genome-wide genotyping, which now amount to little more than a chest x-ray, incorporation of genetic information is achievable for most healthcare systems.

Our premise for pharmacological precision medicine is that by understanding the defects that cause an individual to develop diabetes, overlaid with measures that capture his/her current metabolic state, we will be able to select a best drug for the patient, and this will result in better outcomes for that individual and for the healthcare system. This precision approach would need to be compared with alternative ‘non-precision’ approaches based upon empirical treatment or early use of combination therapy.

## Conclusion: where we are, where we need to go

**Fig. 2:**
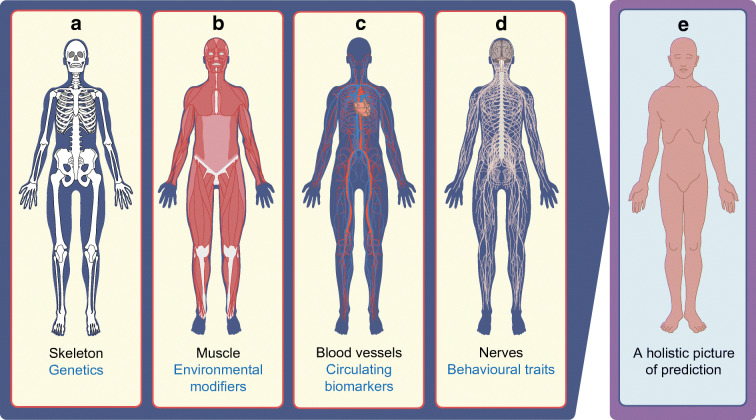
Anatomical analogy of a predictive tool for precision prediction that incorporates relevant axes of biology. (**a**) A robust and reproducible PS denoting a specific diabetes subtype or risk burden would serve as the ‘static’ skeleton, obtained at any point in the individual’s lifetime and signifying its relative immutability. That score would be actualised by robust and reproducible temporal metrics that denote the current developmental and/or metabolic state of the individual, and which could take the form of (**b**) environmental variables (muscle), (**c**) circulating biomarkers (blood vessels) and/or (**d**) behavioural traits (nerves), which together (**e**) offer a holistic picture of prediction. Each of these elements would need to be shown to be robustly associated with clinical outcomes and be cost-effective. This figure is available as part of a downloadable slideset

In our vision, if genomic-based prediction is proven to have sufficient predictive power, it could serve as the scaffolding denoting inherited predisposition to a particular pathogenic process. On this relatively static skeleton, organic modifiers in the form of biomarkers that signify environmental or behavioural factors (the flesh and blood) would complete the predictive picture (Fig. 2). Under this anatomical analogy, the precision roadmap is largely aspirational: of the items that are needed (Fig. 1), we are in the midst of generating the data for Step 1 (genomic predictors) for a handful of type 2 diabetes-relevant medications: this needs to be expanded to all the major drug classes and to include hard endpoints such as cardiovascular outcomes, in large enough sample sizes, across all major populations. We are only beginning to use high-throughput technologies to identify clinically relevant biomarkers (Step 2), and perhaps honing in on those that guide the construction of diabetes subtypes [6]. There are only a few instances whereby these predictive tools might make a clinical difference (Step 3), and studies of cost-effectiveness (Step 4) or venues for implementation (Step 5) remain at the contemplative stage.

To achieve our vision, we must continue to encourage the creation and support of international consortia that foster widespread collaboration, both in terms of the resources needed for them to function, as well as the culture that sees team science as a win–win proposition. Efforts such as the IMI and AMP initiatives that make government–industry–academia cooperation more seamless and fluid should be welcomed, by simplifying regulatory hurdles and encouraging cross-fertilisation. Finally, the earnest engagement of investigators and populations other than those of European descent will be critical to ensure that the tools that emerge can be effectively translated in an increasingly cosmopolitan and inter-dependent world.

## Supplementary information

Slideset of figures (PPTX 359 kb)

## Abbreviations

AMP: Accelerating Medicines Partnership
AMP-CMD: Accelerating Medicines Partnership–Common Metabolic Diseases
AMP-T2D: Accelerating Medicines Partnership–Type 2 Diabetes
CAD: Coronary artery disease
DPP-4: Dipeptidyl peptidase-4
EHR: Electronic health record
GLP-1RA: Glucagon-like peptide-1 receptor agonist
GWAS: Genome-wide association studies
IMI: Innovative Medicines Initiative
NIH: National Institutes of Health
PS: Polygenic score
SGLT2: Sodium–glucose cotransporter 2
