# Supplementary material for: A roadmap to achieve pharmacological precision medicine in diabetes
Source: Diabetologia. 2022 Jun 24;65(11):1830–8. doi: 10.1007/s00125-022-05732-3 (PMC9522818; doi:10.1007/s00125-022-05732-3)
Supplement: Supplementary file 1 — (PPTX 359 kb) [file 125_2022_5732_MOESM1_ESM.pptx]

## Slide 1
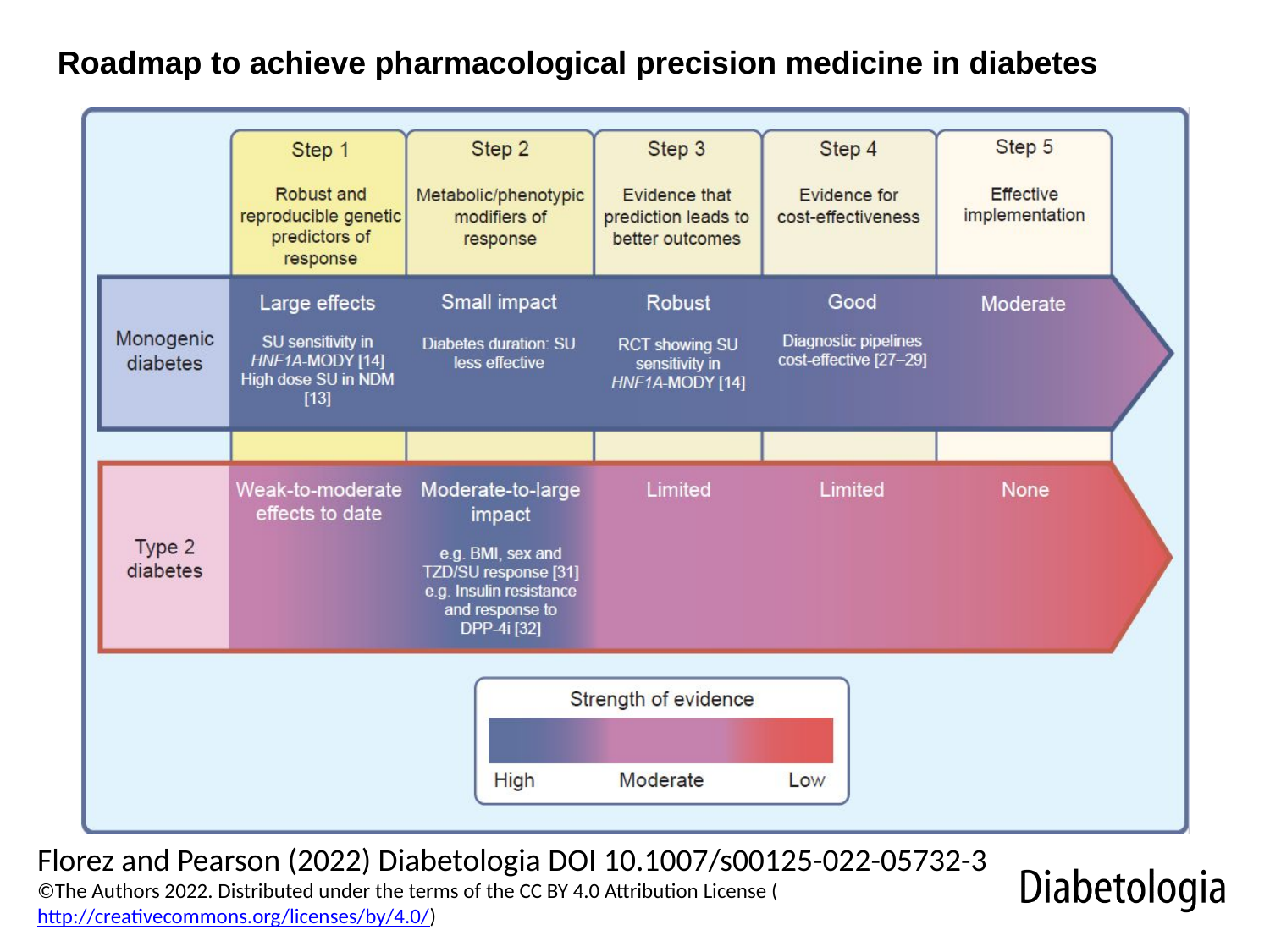

Roadmap to achieve pharmacological precision medicine in diabetes
Florez and Pearson (2022) Diabetologia DOI 10.1007/s00125-022-05732-3
©The Authors 2022. Distributed under the terms of the CC BY 4.0 Attribution License (http://creativecommons.org/licenses/by/4.0/)

## Slide 2
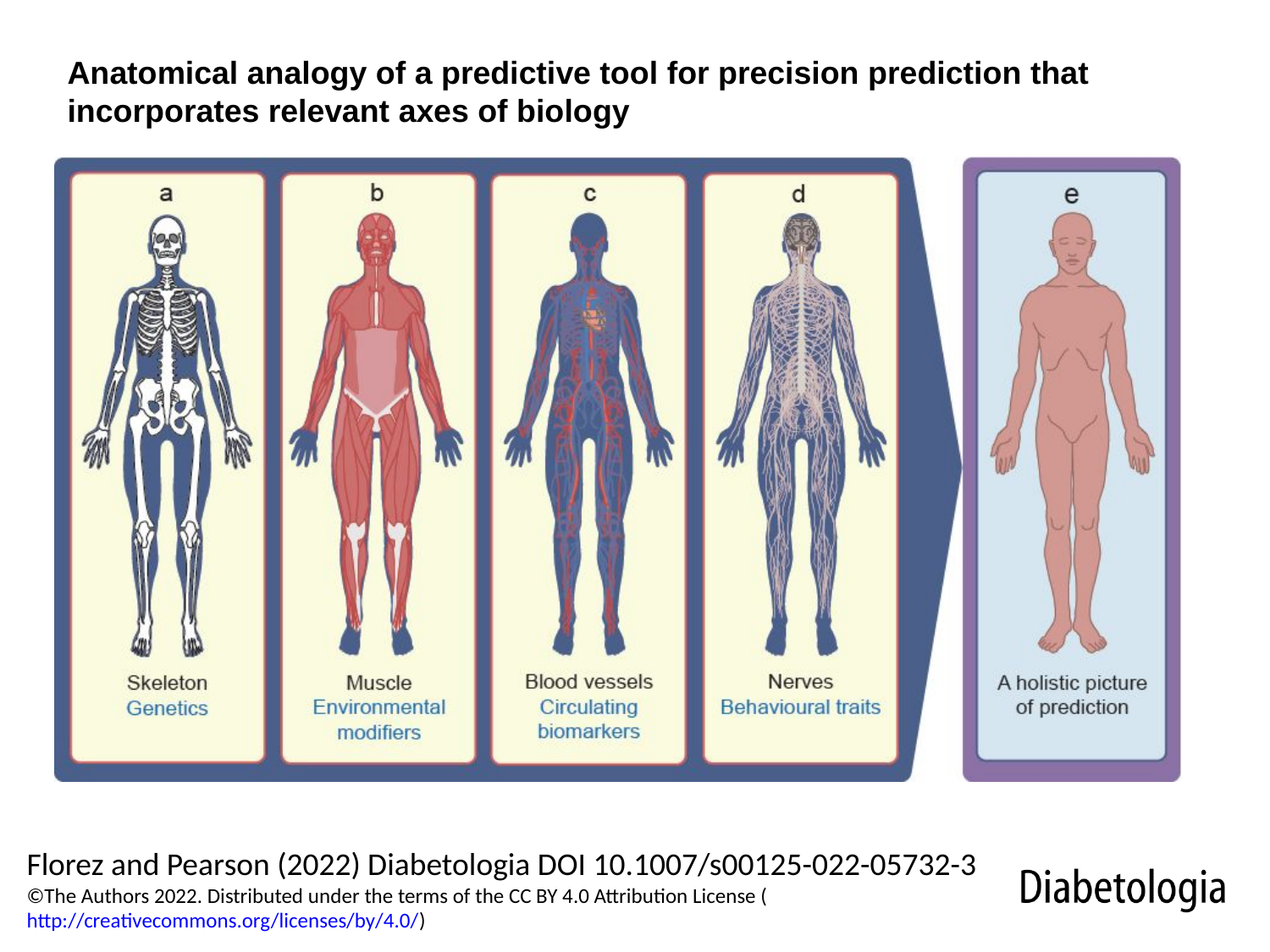

Anatomical analogy of a predictive tool for precision prediction that incorporates relevant axes of biology
Florez and Pearson (2022) Diabetologia DOI 10.1007/s00125-022-05732-3
©The Authors 2022. Distributed under the terms of the CC BY 4.0 Attribution License (http://creativecommons.org/licenses/by/4.0/)
